# Foraging Behaviour and Landscape Utilisation by the Endangered Golden-Crowned Flying Fox (*Acerodon jubatus*), The Philippines

**DOI:** 10.1371/journal.pone.0079665

**Published:** 2013-11-21

**Authors:** Carol de Jong, Hume Field, Anson Tagtag, Tom Hughes, Dina Dechmann, Sarah Jayme, Jonathan Epstein, Craig Smith, Imelda Santos, Davinio Catbagan, Mundita Lim, Carolyn Benigno, Peter Daszak, Scott Newman

**Affiliations:** 1 Queensland Centre for Emerging Infectious Diseases, Department of Agriculture, Fisheries and Forestry, Coopers Plains, Queensland, Australia; 2 EcoHealth Alliance, New York, New York, United States of America; 3 Protected Areas and Wildlife Bureau, Department of Environment and Natural Resources, Quezon City, Philippines; 4 Max Planck Institute of Ornithology, Radolfzell, Germany; 5 Food and Agriculture Organization of the United Nations (FAO), Makati City, Philippines; 6 Department of Agriculture, Quezon City, Philippines; 7 Food and Agriculture Organization of the United Nations (FAO) Regional Office for Asia and the Pacific (FAO RAP), Bangkok, Thailand; 8 Food and Agriculture Organization of the United Nations (FAO), Hanoi, Vietnam; The University of Hong Kong, China

## Abstract

Species of Old World fruit-bats (family *Pteropodidae*) have been identified as the natural hosts of a number of novel and highly pathogenic viruses threatening livestock and human health. We used GPS data loggers to record the nocturnal foraging movements of *Acerodon jubatus*, the Golden-crowned flying fox in the Philippines to better understand the landscape utilisation of this iconic species, with the dual objectives of pre-empting disease emergence and supporting conservation management. Data loggers were deployed on eight of 54 *A. jubatus* (two males and six females) captured near Subic Bay on the Philippine island of Luzon between 22 November and 2 December 2010. Bodyweight ranged from 730 g to 1002 g, translating to a weight burden of 3–4% of bodyweight. Six of the eight loggers yielded useful data over 2–10 days, showing variability in the nature and range of individual bat movements. The majority of foraging locations were in closed forest and most were remote from evident human activity. Forty-six discrete foraging locations and five previously unrecorded roost locations were identified. Our findings indicate that foraging is not a random event, with the majority of bats exhibiting repetitious foraging movements night-to-night, that apparently intact forest provides the primary foraging resource, and that known roost locations substantially underestimate the true number (and location) of roosts. Our initial findings support policy and decision-making across perspectives including landscape management, species conservation, and potentially disease emergence.

## Introduction

Species of Old World fruit-bats (family *Pteropodidae*) have been identified as the natural hosts of a number of novel and highly pathogenic viruses threatening livestock and human health [1]. However, it is increasingly recognised that disease emergence from wildlife is fundamentally an ecological process, often precipitated by anthropogenic activities that result in increased ecological contact between the wildlife, livestock and human ‘systems’ [2]. To understand the drivers for disease emergence from wildlife, and ideally mitigate the risk of emergence, it is necessary to understand the ecology of the natural host, and potential ecological pressures. This knowledge is equally relevant to conservation biology studies.

Detailed movement studies of flying foxes have been limited historically, in part because of their typically high mobility (limiting observational studies) and low bodyweight (limiting remote sensing studies). The cost and weight of telemetry devices have also been factors, however in the last decade, as transmitter size has substantially decreased, satellite telemetry has been increasingly used to describe large scale flying fox movements [3]–[6]. While satellite telemetry is ideal for recording long-range movements of animals, the inherent error associated with location fixes limits detailed movement studies. The alternative Global Positioning System (GPS) technology offers a number of advantages, allowing the capture of detailed and accurate 3-dimensional movement over space and time. While numerous studies have utilised the latter approach to elaborate the detailed movement of avian species [7], [8], its use with bat species is still novel [9], [10]. The approach is particularly attractive where detailed information is sought over a relatively short period of time, and where the biology or behaviour of the study species (such as the colonial diurnal roosting exhibited by flying foxes) facilitates remote downloading of data.

**Figure 1:**
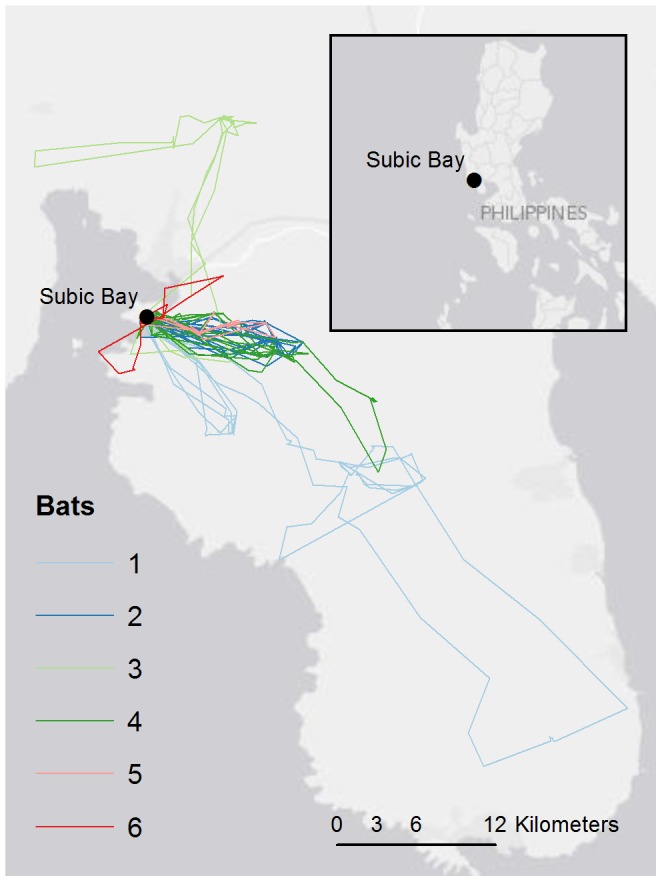
Location of the study and travel routes of all bats obtained during the study. Each bat is represented by one colour.

This study had its origins in the 2008–09 detection of an (asymptomatic) filovirus infection (Ebola Reston virus, REBOV) in domestic pigs [11] and pig workers [12] in the Philippines. In 2010, under the auspices of the Food and Agricultural Organisation of the United Nations (FAO), we undertook virological and ecological studies of a number of bat populations in the Philippines, following identification of species of bats as the natural reservoir of ebola viruses in Africa [13]–[16], Europe [17] and Asia [18]. In this study, we used GPS data loggers to record the nocturnal foraging movements of *Acerodon jubatus*, the Golden-crowned flying fox, on the Philippine’s main island of Luzon (Figure 1). Our aim was to better understand the landscape utilisation of this iconic species, with the dual objectives of pre-empting disease emergence and supporting conservation management. The latter is particularly relevant given that *A. jubatus* is listed as ‘Endangered’ by the International Union for Conservation of Nature (IUCN) (http://www.iucnredlist.org/apps/redlist/details/139/0). The species is also listed as ‘Endangered’ in the Philippine National List of Threatened Wild Fauna, and protected under the Philippine Wildlife Conservation and Protection Act.

## Methods

### Animal Ethics

The study methodology was reviewed and approved by the Philippines National Wildlife Management Committee (NWMC), the body responsible for making recommendations on applications for the use of wildlife for scientific research. Following approval by the NWMC and the local Subic Bay Metropolitan Authority, the Protected Areas and Wildlife Bureau issued a permit (Permit No. 2010-197) for flying fox capture, deployment of data loggers and collection of biological samples. Procedures used for capture, handling, sampling and logger attachment represent current best practice, minimise stress and discomfort, and reduce the risk of injury, mortality and interference to natural behaviour. In addition, flying foxes were captured outside of the breeding and birthing seasons [19] to avoid any negative impact on reproduction, gestation and rearing of young. Further, the study was timed to coincide with favourable environmental conditions and food resource availability to ensure animals were in optimal physical condition. Finally, reconnaissance of the capture site was conducted prior to the study to observe flight patterns and roosting behaviour, minimising disturbance to the colony and roosting habitat during capture.

### Bat Capture

Fifty-four *A. jubatus* were captured by mistnet [20] pre-dawn and post-dusk in the immediate vicinity of a known roost (Roost 1) in the Cubi area of Subic Bay Freeport Zone between November 22 and December 2, 2010, as part of a broader survey of Philippine bats and novel viruses (manuscript in preparation). Captured bats were held individually in cotton pillowcases and transported 3.4 kilometres to Subic Bay Metropolitan Authority (SBMA) *Wildlife in Need Foundation* veterinary clinic for processing. Bats were sequentially anaesthetised using the inhalation agent Isofluorane [21], individual data and morphometrics recorded, and biological samples taken for virus detection. Age (juvenile or adult) was estimated from dentition and the presence or absence of secondary sexual characteristics. Body condition was defined as ‘good’, ‘fair’ or ‘poor’ on the basis of palpation of pectoral muscle mass. Only bats weighing 700 grams or more were considered for deployment of data loggers to limit the logger weight to a maximum 4% of bodyweight [22], and thus minimally impact normal movement behaviour. All bats were recovered from anaesthesia, offered fruit juice (for hydration and energy), and released at their capture location within four hours of capture.

### Data Logger Specifications, Application and Operation

Eight 30 gram GPS/acceleration data loggers (60×25×10 mm) were available for deployment. The loggers were produced by e-obs GmbH (Munich, Germany) and feature programmable GPS, UHF radio and acceleration settings. All loggers were uniquely identified, and programmed with a 900 second interval between GPS fixes, activated between 18∶00 hours and 06∶00 hours daily, enabling up to 48 GPS fixes per animal, per night. Loggers were also programmed to transmit a UHF radio signal between 12∶00 and 14∶00 hours daily. Acceleration data was not included. Projected battery life with this configuration was 15–20 days. Logger memory capacity was 8 megabytes.

**Figure 2:**
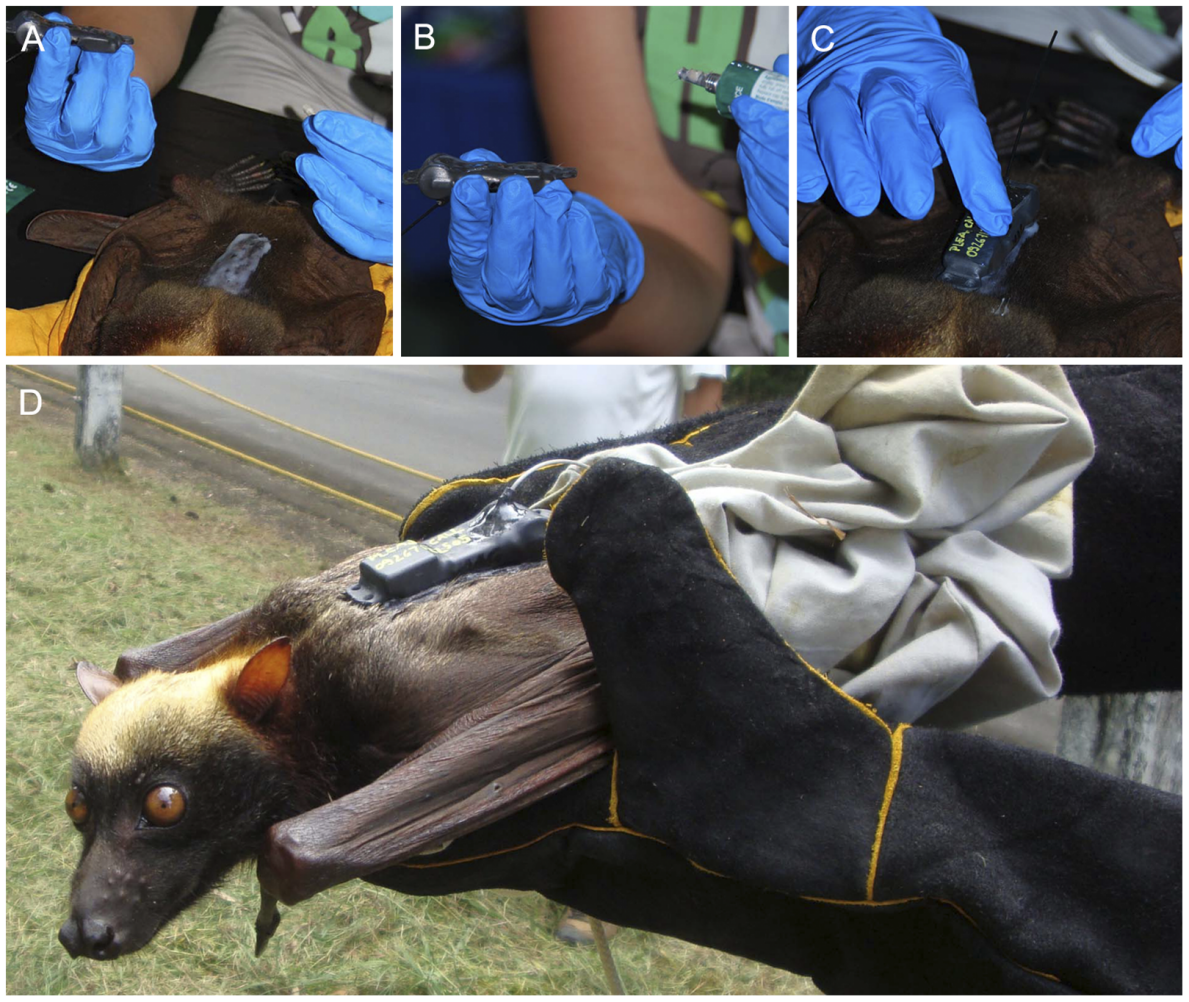
Attachment of GPS data logger. The area distal to the scapulae was clipped to 2–3 mm length (not shown), and skin adhesive (Sauer-Hautkleber™) applied and allowed to semi-cure (A). The base of the logger was coated with 100% ethyl isocyanoacrylate glue (B), and pressed onto the bed of tissue glue, with the aerial trailing caudally (C). The logger was held firmly in place until the glue dried and the bat was released within 2 hours (D).

The logger was glued to the back of the anaesthetised bat, distal to the scapulae, providing an effective and low-impact means of temporary attachment of around 2 weeks. The hair was first clipped to 2–3 mm length, and skin adhesive (Sauer-Hautkleber™) applied to the area and allowed to semi-cure. The base of the logger was coated with 100% ethyl isocyanoacrylate glue, and pressed onto the bed of tissue glue, with the aerial trailing caudally. The logger was held firmly in place until the glue dried, typically in 10–15 minutes (Figures 2a–d). Particular care was taken to avoid excess glue, and to ensure that the logger position did not impede flight movement. Data logger functionality was confirmed, and the bat was removed from anaesthetic and observed until it regained consciousness (typically <5 minutes). The bat was held in its individual bag for a further 0.5–2 hrs prior to release (as described above).

Roost 1, and a second (sometimes occupied) known roost nearby (Roost 4), were visited at least daily from November 26 until 15 December, 2010, and scanned with a modified Yagi aerial attached to a hand-held e-obs base-station. Individual loggers were consecutively detected, and location data remotely downloaded via a wireless radio-link. The estimated limit of successful download was 100–200 meters, though loggers could be detected over a greater distance. Where a logger was not detected at either of the known roosts, we used a UHF receiver strategically in an effort to locate the logger vicinity, and download the data. Accumulated data was periodically up-loaded from the base-station to the Movebank online database (https://www.movebank.org/) for storage and spatial analysis using Google Earth Pro and ArcView.

### Roost and Foraging Locations

A roost was typically defined as a location where the last morning and the first evening data points for one or more individuals overlapped on one or more days. A foraging location was defined as a one-hectare area from which three or more data points were received from a single individual. Radial distance was defined as the direct distance from the originating roost to the point of interest.

### Statistical Analyses

Summary statistics including percentages, ranges and means are used to describe the data.

## Results

**Figure 3:**
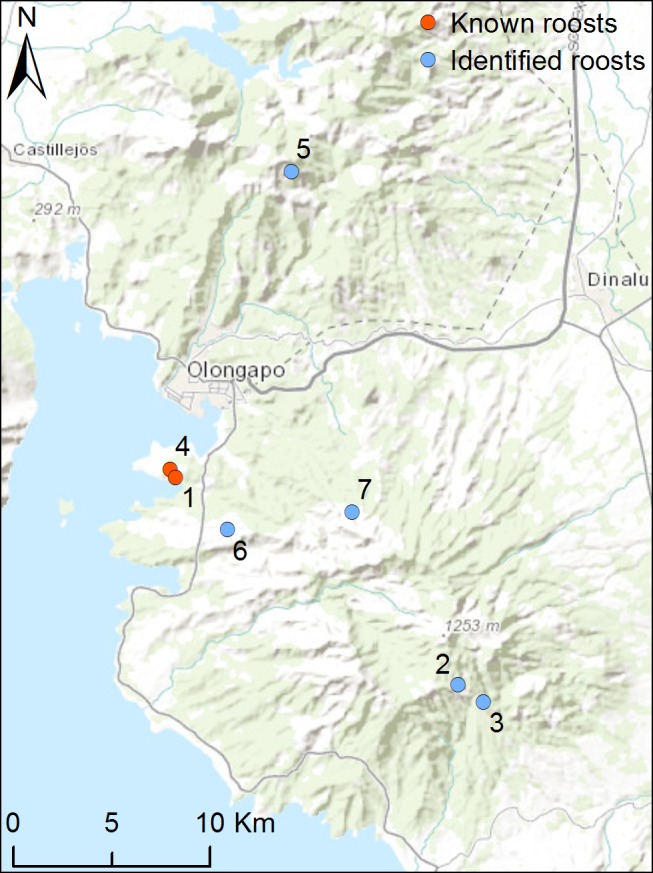
Existing roosts (red markers) and newly identified roosts (blue markers) of *Acerodon jubatus*.

Data loggers were deployed on eight *Acerodon jubatus* on November 25 and December 1, 2010 (Table 1). Bodyweight ranged from 730 g to 1002 g, translating to a weight burden of 3–4% of bodyweight. Individual bat movements were recorded for between two and 10 days. Useable data was collected from six of the eight loggers; no data was received from bat 7, and bat 8 shed the logger at the roost location within hours of deployment. The remaining six loggers were detected either daily or periodically at Roost 1 and Roost 4, or nearby (Figure 3).

**Table 1:** Physical details of individual flying foxes on which GPS data loggers were deployed.

Bat ID	Sex	Age	Weight(g)	Forearm (mm)	BCS
1	M	Adult	764.0	165.0	Good
2	F	Adult	930.0	193.0	Fair
3	F	Adult	740.0	175.0	Good
4	M	Adult	780.0	183.0	Good
5	F	Adult	832.0	177.0	Fair
6	F	Adult	1002.0	196.0	Good
7	F	Juvenile	730.0	180.0	Good
8	F	Adult	928.0	180.0	Good

**Figure 4:**
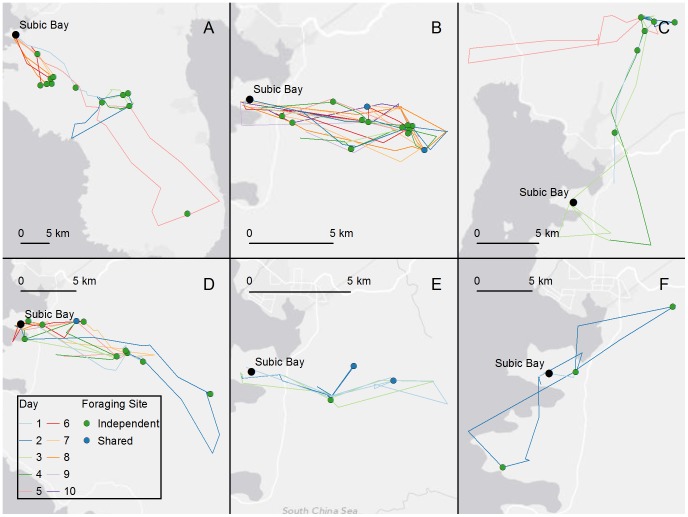
Foraging areas and travel routes of Bats 1–6. Coloured lines represent days. Green markers represent foraging areas used by bats independently. Blue markers indicate foraging areas shared between bats 2 and 4, and bats 4 and 5.

A total of 46 discrete foraging locations, plus five previously unrecorded roost locations (Roosts 2, 3, 5, 6, and 7) were identified (Figures 3 & 4). Roost 4, located 500 m south-east of Roost 1, was previously known. The majority of foraging locations (82.6%) were in closed forest (>100 m from the forest edge), and most were remote from evident human activity (Figure 4a–f). There was variability in the nature and range of individual bat movements (Table 2). Bats 2, 4 and 5 foraged predominantly within an area 12.5 km east-south east of Roost 1 while bats 1, 3 and 6 showed independent foraging behaviours travelling further afield in different directions. A description of the foraging behaviour and landscape utilisation of each individual bat follows, and a visualisation presented in Video S1.

**Table 2:** Movement details of individual flying foxes on which GPS data loggers were deployed^1^.

Bat ID	Date deployed	Nights	Total distance travelled (km)	Mean distance travelled per night (km)	Range - distance travelled per night (km)	Maximum radial distance from release (km)	Total number of foraging areas used	Mean number of foraging areas per night	Mean distance to foraging areas	Range - distance travelled to foraging areas	Mean distance between foraging areas
1	25/11/2010	8	238.35	29.79	9.3–87.04	46.2	12	2	8.9	2.83–14.62	3.4
2	25/11/2010	10	234.32	26.04	19.24–36.52	12.1	15	5	7.9	6.03–10.02	2.7
3	25/11/2010	5	68.90	13.78	5.8–19.5	17	6	3	11.1	1.07–18.19	3.5
4	25/11/2010	8	168.11	21.01	7.89–40.37	20.1	13	4	5.7	1.61–8.28	4.1
5	1/12/2010	3	56.16	18.72	11.72–25.42	9.9	4	3	5.6	3.7–6.8	3.3
6	1/12/2010	2	34.28	17.14	3.88–30.4	6.9	3	2	3.5	1.41–4.17	5.3

1 No useable data was obtained from bats 7 and 8.

### Bat 1

Data was recorded on Bat 1 for eight nights until the battery was depleted, during which time the bat travelled a total of 238.35 km (Table 2). The bat travelled and foraged in a predominantly south-easterly direction, with a maximum 87.04 km return travel one night, contrasting with a minimum 9.3 km the previous night (Figure 4a). It typically utilised new foraging locations each night, and moved between four roosts in the five days following deployment. The first night after deployment, it relocated 18 km south east to Roost 2 where it remained for 2 days, before moving a further 1.3 km south east to Roost 3 for one day. It then relocated to Roost 4, where it remained for two days, before returning to Roost 1 on day 7.

### Bat 2

Data was collected on Bat 2 over 10 nights until the logger memory was full, although the battery lasted 19 days (Table 2). This bat remained at Roost 1 for the entire first night, then travelled a total of 234.32 km over the following nine nights. It used two roosts, based at Roost 1 for the first three days, then relocating to Roost 4 for two days before returning to Roost 1. Travelling and foraging was in a localised area east-south-east of Roost 1 (Figure 4b). Bat 2 followed an almost identical route from the roost on nights 2, 3 and 4, and an almost identical path on the return flight every night. It utilised a total of 15 foraging locations, visiting an average of five per night, and re-visiting the same locations on five consecutive nights. Foraging behaviour was characterised by frequent switches between foraging locations; for example night 4, where it switched between three locations 15 times over a 7.5 hour period.

### Bat 3

Bat 3 was not detected at Roost 1 or Roost 4 during the entire study period. The logger was located 10 days after deployment, when a weak UHF radio signal revealed its location, 1.6 km east of Roost 1. It had recorded five nights of movement before being shed. The bat travelled a total of 129.78 km in that time (Table 2). Roosting and foraging activity were predominantly to the north of Roost 1 (Figure 4c). The bat utilised six foraging locations, however most foraging activity occurred at just one location within 1 km of Roost 5. The remaining locations were visited either en route to the north, or intermittently for short periods over successive nights. Roost 5, 16.4 km north of Roost 1, was used for all but one of the five days, when the bat returned to the vicinity of Roost 1, before roosting 4.3 km to the south-east at Roost 6. The following night, it returned to the north to forage, roosting again at Roost 5. The final night of data revealed novel behaviour with the bat conducting a 28 km return flight to the west, exhibiting erratic flight patterns for a brief period on return to the foraging location of the previous nights.

### Bat 4

The movements of Bat 4 were recorded for eight nights; data points for the subsequent two nights were clustered just to the north-east of Roost 1, suggesting the logger was shed shortly after the bat left the roost on Day 9. The logger remained active for 19 days until the battery was depleted. During the eight-day period, the bat travelled a total distance of 168.11 km (Table 2) (Figure 4d). Foraging was focused in an area to the east of Roost 1, with the exception of night 2 when the bat travelled a further 10 km to the south-east. Travelling and foraging were predominantly in the same area as Bat 2, but Bat 4 utilised 13 separate foraging locations, two of which were also utilised by Bat 2. Bat 4 utilised three roosts within five days, leaving Roost 1 after deployment to roost 9.4 km south east at Roost 7. It returned to Roost 1 the following morning for one day, then relocated to the Roost 4, shared by Bats 1 and 2 thereafter, returning to Roost 1 on the morning of day nine.

### Bat 5

The movements of Bat 5 were recorded over three nights, after which the bat was not detected again. The total distance travelled was 56.16 km (Table 2) (Figure 4e). Foraging and travel were focused to east/south-east of Roost 1, where the bat utilised a total of four separate foraging locations. The same foraging location was utilised first each evening for several hours before the bat moved to other locations which were also visited by Bats 2 and 4. The bat returned to Roost 1 each morning.

### Bat 6

The movement activity of Bat 6 was recorded over two nights, and the bat was last detected at Roost 1 on day 3. It travelled a total 34.28 km (Table 2) (Figure 4f). The bat spent the entire first night at one foraging location 4 km east of Roost 1. The second night, it travelled a total of 30.4 km, taking an indirect route (to the north) to forage in the same location as the previous night. It then travelled a direct line 5.2 km further north to forage for a short period, before again returning to the earlier location. The bat then travelled an indirect route 5 km to the south to forage briefly before returning to Roost 1. Bat 6 did not use any other roosts, returning to Roost 1 on both mornings.

## Discussion

Flying foxes are typically a nomadic species whose movements are primarily driven by food resource availability [19]. Historic food resources are increasingly threatened by extreme weather events, changing climatic ‘norms’ and anthropogenic impacts [19], with potential negative consequences for both bat and man. The IUCN lists *A. jubatus* as ‘Endangered’, with an estimated 50% population decline over the last in thirty years, or three generations (http://www.iucnredlist.org/apps/redlist/details/139/0). Thus, understanding the foraging movements and landscape utilisation of *A. jubatus* informs landscape conservation and management strategies, biodiversity strategies, and potentially emerging disease risk management.

While the study involved a limited number of bats, a number of relevant findings have been made. Firstly, foraging is not a random event. The majority of bats exhibited night-to-night repetition of movements, indicating that once a food resource was located, repeat utilisation occurred. This is intuitively more energy-efficient that random foraging, and putatively provides a more constant daily nutritional intake. However, in the context of emerging disease risk, repetitious foraging behaviour by a flying-fox that is excreting virus means a cumulative viral load under the feed tree/trees, which both prolongs the exposure risk period and potentially facilitates an infectious dose of virus. Bat 2 appears to have taken foraging site re-visits to an energy-sapping extreme, moving between three sites 15 times on night 4 (Figure 4b). Albeit that the average distance between the three sites was only 2.7 km, the behaviour seems energetically flawed. One plausible explanation for the behaviour could be periodic disturbance at one or more of the sites, or the bat monitoring flowers for peak nectar flow [23]. Bat 3 exhibited a period of apparently erratic movement on return to roost on night five (Figure 4c). Examining the terrain at this point, the behaviour may be explained by the bat negotiating a ridgeline, with it initially flying almost around the end of the ridge, before turning to fly back along the ridgeline for 2 km, then up a gully line and over the ridge. Bat 1 also exhibited intriguing behaviour, making a 165.24 km return journey from Cubi over 5 nights. It is tempting to interpret this movement behaviour as long-range foraging reconnaissance, and if our study cohort broadly reflects the population structure, it may indicate that 20% of bats periodically make such movements, which may predicate colony movement at a regional or inter-regional level.

A second relevant finding relates to the predominance of foraging sites in closed forest habitat. The majority of these were in apparently intact forest, remote from evident human activity; a minority were in forest fringing farmland or peri-urban areas. This suggests that (at least at the time of the study) the majority of food resource available to flying foxes in the region occurred within the undisturbed closed forest habitat. This landscape utilisation is in stark contrast to the peri-urban location of the Roosts 1 and 4; both are in the immediate vicinity of a periodically busy local road (the former straddling the road). Thus, while intact forest habitat is not a prerequisite for a roost site, it is evidently essential for successful foraging and food resource availability. This finding has major implications for species conservation and for emerging disease risk, in terms of managing agricultural and urban encroachment. Other authors have also noted the foraging preference of *A. jubatus* for primary forest, and the conservation threat posed by forest disturbance [19], [24]. From an emerging disease perspective, foraging in primary forest means that the opportunity for contact between flying-foxes and humans and livestock species (and thus emerging disease risk) is reduced. While *A. jubatus* have not specifically been associated with emerging zoonoses, Breed et al (2013) [25] recently reported henipavirus infection in *A. celebensis* in the neighbouring Indonesian island of Sulawesi. More broadly, the demonstrated association of multiple megachiropteran species with henipaviruses, SARS-related coronaviruses and ebola viruses [1] across a geographic spectrum suggests that such an association is probable. Studies of related *Pteropus* species in Australia, Malaysia and Bangladesh have demonstrated how an understanding of the ecology of the host species can inform an understanding of the ecology of emerging zoonotic viruses.

A third significant finding relates to the identification of previously unrecorded roost locations. We identified five ‘new’ roost locations, four of them in inaccessible forested locations, precluding ‘ground-truthing’. While on occasion, weak and starving flying foxes have been observed to roost close to scarce food resources, presumably in an effort to conserve dwindling energy reserves (H. Field, unpublished data), the positive body condition of flying foxes in the study is not consistent with this scenario. Further, a number of the roost locations (Roosts 2, 4 and 5) are used as a base over multiple nights, with the individual making foraging forays from the site each evening, and returning to the site the next morning. This behaviour is consistent with the site being an established roost. Ground-truthing of the fifth putative new roost (Roost 6) more than 12 months later found no flying foxes present at that time, but identified a very large towering tree locally called Kupang (*Parkia timoriana*), the same species utilised at known Roosts 1 and 4. There were also abundant *Ficus* sp. (a seasonal food resource) along a nearby creek. Disconcertingly, the presence of wildlife traps in the vicinity indicates that the area is used by hunters.

While our study cohort had a female bias, the six bats from which we obtained useable data comprised two males and four females. One of the four females was pregnant on abdominal palpation, though it is possible the other females were at an earlier stage of gestation and the fetus not palpable. We made a conscious decision to include pregnant and possibly pregnant females in the cohort, with the knowledge that the loggers were typically shed within two weeks. This meant that logger weight/bodyweight ratio did not need to be the sole criteria for deployment. This was a major advantage of the GPS data-loggers over satellite telemetry, and allowed us to study the foraging movement behaviour of this typically excluded cohort. Nonetheless, all the deployed loggers in the study weighed less than 4% of bat bodyweight.

Two instrumented bats failed to yield useful data: Bat 7, released the evening of December 1, was not detected subsequently. Given that the logger functionality was confirmed at fitting, the most plausible scenario is that the bat left Roost 1 and the area that evening, and did not return during the (limited) remainder of the study period. Bat 8 appears to have shed its logger at Roost 1 within 24 hrs of deployment, indicated by the clustering of all subsequent data points within the roost. This suggests either faulty attachment of the logger, premature release of the bat (before the glue had fully cured), or removal of the logger by the bat.

A fundamental limitation of current GPS data loggers is the necessity to subsequently locate instrumented individuals to recover logged data. While the colonial roosting behaviour of *A. jubatus* facilitates this, their mobility and underlying nomadic behaviour can undermine it, and active and determined efforts are needed to maximise data recovery. In this study, the radiotelemetry capability of our loggers supported this effort, and allowed us to locate and download data from Bat 3 that we would have otherwise not recovered.

## Conclusion

We used GPS data loggers to study the foraging behaviour and landscape utilisation of *Acerodon jubatus* on the Philippine Island of Luzon. The GPS data logger technology, our strategic ‘duty cycle’ program, our deployment of the loggers, and our recovery of the data allowed us to meet this aim. Six of eight deployed loggers returned detailed data on the foraging and roosting behaviour of this IUCN-listed ‘Endangered’ species: firstly, that foraging is not a random event, with the majority of bats exhibiting night-to-night repetition of movements, indicating that once a food resource is located, repeat utilisation continues while the resource lasts; secondly, that the majority of foraging sites were in apparently intact forest, remote from evident human activity, suggesting that this natural resource is essential for successful foraging and food resource availability; and thirdly, we identified five previously unrecorded putative roost locations in remote forest habitat, that with ground validation, will significantly expand knowledge of the regional landscape utilisation by this species. While future targeted studies could augment this study, our findings usefully inform policy and decision-making across perspectives including landscape management, species conservation, and potentially disease emergence.

Finally, the GPS data logger approach has a clear role in movement studies of flying foxes, with the ability to provide accurate data on roosting, foraging and broader nomadic movements across the landscape. The level of detail complements the ‘bigger picture’ perspective offered by satellite telemetry, and offers an exciting insight into future advancement of the technology.

## Supporting Information

Video S1 ***Acerodon jubatus*** **movement.**
(MP4)
