# Correction: Foraging Behaviour and Landscape Utilisation by the Endangered Golden-Crowned Flying Fox (*Acerodon jubatus*), The Philippines

**DOI:** 10.1371/annotation/67f7a1ef-d1bb-4752-a44e-beaca05a126b

**Published:** 2013-12-03

**Authors:** Carol de Jong, Hume Field, Anson Tagtag, Tom Hughes, Dina Dechmann, Sarah Jayme, Jonathan H. Epstein, Craig Smith, Imelda Santos, Davinio Catbagan, Mundita Lim, Carolyn Benigno, Peter Daszak, Scott Newman

The name of the seventh author was given incorrectly. The correct name is: Jonathan H. Epstein. 

