# Correction: Foraging Behaviour and Landscape Utilisation by the Endangered Golden-Crowned Flying Fox (*Acerodon jubatus*), The Philippines

**DOI:** 10.1371/journal.pone.0120945

**Published:** 2015-03-26

**Authors:** The PLOS ONE Staff

## Notice of Republication

This article was republished on February 11, 2015, due to the release of confidential or copyrighted material. Please download this article again to view the corrected version.
